# Self-rated health and associated factors among the oldest-old: results from a cross-sectional study in Sweden

**DOI:** 10.1186/s13690-020-0389-2

**Published:** 2020-02-03

**Authors:** Bo Simonsson, Anu Molarius

**Affiliations:** 1 Competence Centre for Health, Region Västmanland, Västerås, Sweden; 2 Centre for Clinical Research, Region Värmland, Karlstad, Sweden; 3 Department of Public Health Sciences, Karlstad University, Karlstad, Sweden

**Keywords:** Oldest-old, Health behaviour, Social factors, Self-rated health, Population studies

## Abstract

**Background:**

Very few population-based studies have investigated self-rated health and related factors in the increasing age group 85 years or older. The aim of this study was to examine self-rated health and its association with living conditions, lifestyle factors, physical and mental health problems and functional ability among the oldest-old in the general population in Sweden.

**Methods:**

The study is cross-sectional and based on 1360 persons, 85 years of age or older, who answered a survey questionnaire sent to a random population sample in 2012 (participation rate 47%). Multivariate logistic regression was used as the statistical method.

**Results:**

The prevalence of good self-rated health was 39% in men and 30% in women. Physical inactivity, impaired physical mobility, pain, anxiety/depression and longstanding illness were independently associated with poorer than good self-rated health, while factors such as gender, age, educational level, cash margin, living alone, social support, smoking, alcohol use, obesity, accidents and impaired vision/hearing were not.

**Conclusions:**

While a considerable part of the oldest-old assess their health as good, not being physically active and having common health problems such as pain and depression as well as impaired physical mobility are associated with poorer than good self-rated health. This should be considered when planning how to improve and maintain health in the growing population of persons 85 years and older.

## Background

Sweden has, as well as other European countries, an ageing population [1]. The proportion of people 85 years or older in Sweden is presently 2.6% and is predicted to increase to 6.8% of the total population in 2030. The average life expectancy is predicted to be 85 years for men and 87 years for women in 2030 [2]. The costs and burden on healthcare and social systems associated with aging will therefore increase in the future.

Getting older does not necessarily mean poorer health and quality of life. Possibilities to influence the health of the elderly are larger than previously thought, and health promotion and prevention activities throughout life, even into advanced age, have positive effects on health and quality of life [3, 4]. The proportion of older people who assess their general health as good or very good has increased in Sweden, but it is mainly the younger pensioners who report better health [5]. Similar findings have been observed in Germany [6].

Self-rated health (SRH) is a self-assessment of an individual’s current health status and one of the most widely used survey measures in medical and social science. SRH has been shown to be associated with multiple dimensions of current and future health - including morbidity [7], functional health [8, 9], and mortality [10, 11], as well as health care utilization and consumption of medication [12]. SRH is strongly associated with a number of determinants that reflect the individual’s living conditions, such as economic hardship and lack of social support, and lifestyle habits such as smoking and physical inactivity [13, 14].

Factors that affect SRH in the elderly include chronic diseases, and physical and mental health [7, 15, 16]. In addition, functional ability has been found to be an important determinant of SRH [8, 9]. Lifestyle factors such as physical activity and smoking have been shown to be associated with SRH even in the elderly [17–19]. Some studies have reported that socioeconomic differences in SRH persist in very old age [20] whereas others have found that this effect decreases with age [9]. In the oldest old, previous studies have investigated the level [21] or trends in SRH [6], socioeconomic differences in SRH [9, 20], associations between SRH and health problems and functional status [8, 9, 22, 23] and included educational level, income, marital status and social support as contributing factors [23]. But studies covering a broader range of contributing factors, including lifestyle factors, in this age group are, to our knowledge, lacking. Thus, there is a need for more population-based studies on SRH and different types of contributing factors specifically in the increasing age group of 85 years or older.

The aim of this study was to explore the prevalence of good/poor SRH, and the relationships between SRH and living conditions, lifestyle factors, physical and mental health problems, accidents and functional ability among the oldest-old in the general population in Sweden.

## Methods

This study is based on data from the survey “Health on equal terms” conducted in 2012 in collaboration with the Swedish Institute of Public Health. The national survey is carried out every year since 2004 to monitor the health of the population in Sweden. The age group addressed is 16–84 years. The sample frame is the total population register at Statistics Sweden, the statistical administrative authority in Sweden, covering all inhabitants in Sweden.

The present study is based on data from two counties (Västmanland and Uppsala) where the questionnaire was sent during April – June 2012 also to 2870 persons in the age group 85+ years, of which 1360 answered the questionnaire (response rate 47%). The sample was random and stratified by gender and municipality. The questionnaire could also be responded online, but more than nine in ten respondents in this age group used the postal questionnaire. Data collection was discontinued after two postal reminders. The mean age of the respondents was 88.6 years among women and 88.2 years among men.

Information on gender, age, level of education and country of birth is based on register data from Statistics Sweden. For those not covered in the education register the survey responses to the question on educational level were used. The information letter included a comment that the respondent should feel free to ask for help from a person close to one in case he/she had difficulties in filling in the questionnaire himself/herself.

### Outcome

*Self-rated health:* Was measured with the question “How would you assess your general state of health?” Response options were: Very good, Good, Fair, Poor and Very poor. The two first response options were regarded as good and the two last options as poor SRH. In the statistical analysis the options were dichotomised into good and poorer than good SRH.

### Living conditions

*Educational level* was categorised into three levels: compulsory education, secondary education and post-secondary education.

*Country of birth* was dichotomized into those born in Sweden and those born outside Sweden.

*Living alone* was measured by one question “Who do you share a home with?” where the response option Nobody was coded as living alone.

*Cash margin* was measured with the question “If you should suddenly find yourself in an unforeseen situation where you had to find 15 000 SEK in one week, would you manage it?” (Yes/No).

*Social support* was measured with the question “Do you have anyone you can share your innermost feelings with and confide in?” (Yes/No).

### Lifestyle factors

*Physical activity:* To measure physical activity, two questions were used originating from the International Physical Activity Questionnaire (IPAQ) [24]. The first question was “How much physical movement and exertion have you had in the last 12 months?” The response options were: Sedentary lifestyle, Moderate exercise in leisure time, Moderate, regular exercise in leisure time and Regular exercise and training, with descriptions of needed activity for each level. The second question regarded activity during a typical week: “How much time do you spend in a normal week in moderately strenuous activities that make you warm?” The answer options were: 5 h or more a week, More than 3 h a week and less than 5, Between 1 and 3 h a week, No more than 1 h a week, and Not at all. To be considered physically active (at least 30 min per day) in this study required that the respondent answered option three or four on the first question or option one or two in the second.

*Smoking habits* were measured by a question “Do you smoke every day?” (Yes/No).

*Alcohol habits* were measured by a question “How often have you drunk alcohol in the last 12 months?” Response options were: 4 times a week or more, 2–3 times a week, 2–4 times a month, Once a month or less and Never.

*Obesity* was categorized after calculating body mass index (BMI) obtained from the participant’s self-reported height and weight. Participants were categorized into obese (BMI ≥ 30 kg/m^2^) and not obese (BMI < 30 kg/m^2^) [25].

### Health problems

*Accidents* were measured by the question “Have you had any accidents in the last three months that led to your seeking health care or dental care?” The response options were: No, Yes, once and Yes, more than once.

*Impaired vision* was measured with the question “Can you see and make out normal text in daylight without difficulty?” Response options were: Yes, without glasses, Yes, with glasses and No. The last response option was coded as impaired vision.

*Impaired hearing* was measured with the question “Can you hear what is being said in a conversation between several persons without difficulty?” Response options were: Yes, without a hearing aid, Yes, with a hearing aid and No. The two last options were coded as impaired hearing.

*Physical mobility:* To measure physical mobility the questions used were “Can you walk up steps without difficulty (for example steps up to a bus or train)?” and “Can you take a short walk (about five minutes) at a reasonably fast pace?” The response options were: Yes and No. The participant was considered to have physical mobility if he/she answered yes to both questions.

European Quality of life (EQ-5D-3 L) questionnaire is a standardized instrument that measures health outcomes [26]. The instrument has five dimensions: mobility, self-care, usual activities, pain/discomfort and anxiety/depression.

*Pain* and *Anxiety/depression:* To measure pain the fourth dimension and to measure anxiety/depression the fifth dimension in the EQ-5D-3 L scale was used, with the three levels: No problems, Some problems and Extreme problems. The two last levels were coded having pain or anxiety/depression, respectively.

*Longstanding illness* was used to measure chronic conditions among the elderly and was assessed with the question: “Do you have any long-term illness, discomfort following an accident, any reduced physical function or any other long-term health problem?” (Yes/No).

### Ethical considerations

The study followed the Swedish guidelines for studies in social sciences and humanities, in accord with the Declaration of Helsinki and the data are protected by the law of official statistics. The participants were informed that completed questionnaires would be linked to the Swedish official registries through personal identification numbers, to access registry information on gender, age, country of birth and educational level. The respondents thus gave their informed consent to the linking of registry data. Immediately after the record linkage, the personal identification numbers were deleted. Statistics Sweden carried out the sampling, data collection and linkage with registry data and delivered the de-identified data to the County Councils. The survey was approved by the Regional Board of Ethics, Uppsala (EPN 2012/256).

### Statistical analysis

Differences in the distribution of background characteristics and SRH between men and women were tested using chi-square statistics. *P*-values < 0.05 were considered statistically significant. The relationships between living conditions, lifestyle habits, accidents, functional ability and health problems in relation to SRH were studied using bivariate and multivariate logistic regression. The results are reported as odds ratios (OR) and 95% confidence intervals (95% CI). All analyses were conducted in IBM SPSS Statistics, version 20.

## Results

Most of the participants in this study had compulsory or secondary education and were born in Sweden (Table 1). More women than men reported that they are living alone: seven out of ten women and four out of ten men. Only one in ten of both men and women indicated that they had no or little social support and that they lacked cash margin. Four out of ten men were physically active at least 30 min per day or more, compared to two out of ten women. The prevalence of alcohol use was higher among men than among women, while smoking was rare among both men and women.

**Table 1:** Background characteristics of women and men aged 85 years and older

	Women	Men	Total	*P*-value for difference between men and women^a^
N	592	768	1360	
Living conditions				
Educational level (%)				
Low	60.9	50.5	55.0	**< 0.01**
Medium	25.6	30.2	28.2	
High	13.6	19.3	16.8	
Country of birth (%)				
Sweden	90.0	92.4	91.4	0.12
Other	10.0	7.6	8.6	
Living alone (%)				
Yes	72.0	37.9	52.7	**< 0.01**
No	28.0	62.1	47.3	
Cash margin (%)				
Yes	86.2	92.0	89.5	**< 0.01**
No	13.8	8.0	10.5	
Social support (%)				
Yes	85.0	87.3	86.3	0.25
No	15.0	12.7	13.7	
Lifestyle habits				
Physical activity (%)				
Yes	24.3	39.4	33.0	**< 0.01**
No	75.7	60.6	67.0	
Alcohol use 4 times/week or more (%)				
Yes	4.0	7.9	6.2	**0.01**
No	96.0	92.1	93.8	
Smoking daily (%)				
Yes	2.4	2.0	2.2	0.68
No	97.6	98.0	97.8	
Obesity (%)				
Yes	11.5	8.6	9.8	0.09
No	88.5	91.4	90.2	
Health problems				
Accident last 3 months (%)				
Yes	10.7	10.7	10.7	0.97
No	89.3	89.3	89.3	
Impaired vision (%)				
Yes	15.0	11.3	13.0	**0.05**
No	85.0	88.7	87.0	
Impaired hearing (%)				
Yes	55.3	59.9	57.9	0.09
No	44.7	40.1	42.1	
Physical mobility (%)				
Yes	44.3	56.5	51.4	**< 0.01**
No	55.7	43.5	48.6	
Pain (%)				
Yes	80.3	75.1	77.4	**0.03**
No	19.7	24.9	22.6	
Anxiety/depression (%)				
Yes	36.9	26.3	30.9	**< 0.01**
No	63.1	73.7	69.1	
Longstanding illness (%)				
Yes	59.8	56.4	57.9	0.23
No	40.2	43.6	42.1	

^a^Statistically significant differences marked with bold

Health problems were common in this age group (Table 1). Majority of both men and women reported at least some problems with pain and six out of ten indicated that they had longstanding illness. About half of the respondents had impaired physical mobility. Impaired vision, impaired physical mobility, pain and anxiety/depression were more common in women than in men.

**Fig. 1:**
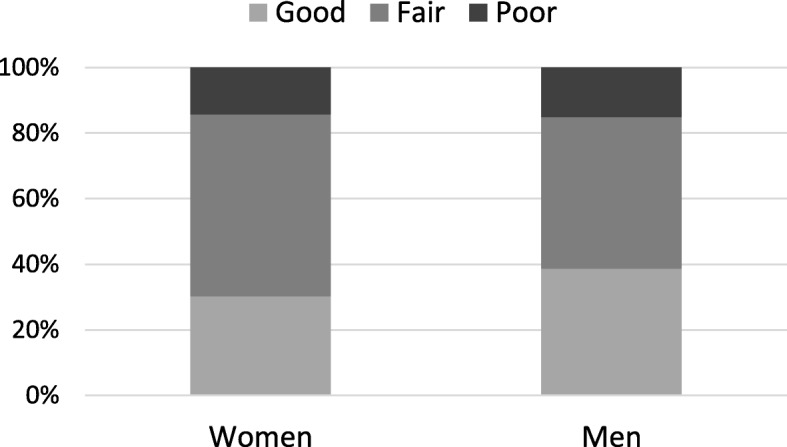
Distribution of self-rated health (%) among women and men aged 85 years and older (*N* = 1360)

The proportion of women who rated their health as good was 30%, while the proportion in men was 39% (Fig. 1) (*p* = 0.003). More women than men rated their health as fair, whereas the proportion with poor SRH was similar in both women and men, 14–15%.

In the bivariate model the odds ratio for poorer than good SRH was statistically significant for female gender, low educational level and lack of social support (Table 2). In addition, physical inactivity and obesity were significantly associated and alcohol use had an inverse association with poorer than good SRH. All included health problems i.e. accidents, impaired vision, impaired hearing, physical mobility, pain, anxiety/depression and longstanding illness had odds ratios that were statistically significantly higher than 1. Age, country of birth, living alone, cash margin and smoking were not statistically significantly associated with SHR.

**Table 2:** Odds ratios (95% confidence intervals in brackets) for poorer than good self-rated health

	OR1^a^	OR2^a^
Age		
85–87	1 (ref.)	
88–99	1.2 (0.9, 1.5)	
Gender		
Man	1 (ref.)	1 (ref.)
Woman	**1.4 (1.1, 1.8)**	1.2 (0.8, 1.7)
Living conditions		
Educational level		
Low	**1.5 (1.1, 2.1)**	1.6 (1.0, 2.5)
Medium	1.3 (0.9, 1.8)	1.6 (0.9, 2.6)
High	1 (ref.)	
Country of birth		
Sweden	1 (ref.)	
Other	1.3 (0.8, 1.9)	
Living alone		
Yes	1.2 (1.0, 1.6)	
No	1 (ref.)	
Cash margin		
Yes	1 (ref.)	
No	1.2 (0.8, 1.7)	
Social support		
Yes	1 (ref.)	1 (ref.)
No	**1.6 (1.1, 2.3)**	1.4 (0.8, 2.6)
Lifestyle habits		
Physical activity		
Yes	1 (ref.)	1 (ref.)
No	**2.8 (2.2, 3.7)**	**1.5 (1.0, 2.2)**
Alcohol use 4 times/w or more		
Yes	**0.6 (0.4, 0.9)**	0.7 (0.4, 1.3)
No	1 (ref.)	1 (ref.)
Smoking daily		
Yes	1.1 (0.5, 2.4)	
No	1 (ref.)	
Obesity		
Yes	**1.6 (1.0, 2.6)**	1.4 (0.7, 2.7)
No	1 (ref.)	1 (ref.)
Health problems		
Accident last 3 months		
Yes	**1.8 (1.2, 2.7)**	1.1 (0.6, 2.1)
No	1 (ref.)	1 (ref.)
Impaired vision		
Yes	**2.9 (1.9, 4.5)**	1.5 (0.9, 2.7)
No	1 (ref.)	1 (ref.)
Impaired hearing		
Yes	**1.5 (1.2, 1.9)**	1.0 (0.7, 1.5)
No	1 (ref.)	1 (ref.)
Physical mobility		
Yes	1 (ref.)	1 (ref.)
No	**6.5 (4.9, 8.6)**	**2.7 (1.8, 3.9)**
Pain		
Yes	**6.4 (4.7, 8.6)**	**3.8 (2.5, 5.8**)
No	1 (ref.)	1 (ref.)
Anxiety/depression		
Yes	**5.1 (3.7, 7.1)**	**2.7 (1.8, 4.2)**
No	1 (ref.)	1 (ref.)
Longstanding illness		
Yes	**5.8 (4.5, 7.5)**	**3.3 (2.3, 4.8)**
No	1 (ref.)	

OR1: Bivariate odds ratios

OR2: Adjusted model including statistically significant variables in OR1

^a^Statistically significant odds ratios marked with bold

In the second model where the statistically significant variables were mutually adjusted for, the odds ratios for gender, educational level, social support, alcohol use, obesity, accidents, impaired vision and impaired hearing were no longer statistically significant (Table 2). Physical inactivity, impaired physical mobility, pain, anxiety/depression and longstanding illness remained statistically significantly associated with poorer than good SRH in the adjusted model.

## Discussion

There was a difference between men and women in the distribution of SRH, 39% of the men rated their health as good compared to 30% of the women. However, about 15% among both women and men rated their health as poor. The finding that men have better SRH than women is in line with several other studies [17–19, 27]. In most studies younger elderly have been found to have better SRH than older elderly [16, 19, 27] whereas in a study of elderly in Spain the opposite was found [9]. We did not find any difference in SRH between age-groups 85–87 and 88–99 years. For sensitivity analysis we also tested age groups 85–89 and 90 years and older but there was no difference in SRH (not shown). The relative stability found in SRH may indicate that with increasing age older people adapt to their worsening health condition [17] or it may be due to higher mortality and higher non-response among the participants with poorest SRH [28]. Yet, relatively high proportions of good SRH among the oldest-old have even been reported in other studies [19, 21, 23].

SRH has in previous studies shown to have an independent effect on the risk of future morbidity and premature death [10, 11]. It is also recommended to measure health in populations [29]. However, SRH is a subjective assessment combining different dimensions of an individual’s health and the interpretation may be modified by age [10]. In both cross-sectional and longitudinal studies, lower scores in SRH among elderly appear to be related to three broad factors; number of medical conditions or symptoms [8, 9, 16], functional ability [8, 9], and mental health [22]. We did not have data on specific medical conditions such as cardiovascular disease or dementia, but we used longstanding illness as a measure of chronic conditions. Longstanding illness was strongly associated with SRH in our study, even when other health complaints such as pain and anxiety/depression were taken into account.

Incidence of impaired physical mobility increases with age [5]. Some previous studies have investigated physical mobility and SRH and have found an association [15, 23]. There was a strong association between impaired physical mobility and SRH in our study, which highlights the importance of improving and maintaining physical mobility among the oldest-old. The interpretation of functional decline in very old age is, however, complex [30].

Anxiety/depression was strongly associated with poorer than good SRH in our study which corroborates with findings from other studies [7, 22, 31]. Mulsant et al. [31], for example, found that depressive symptoms were strongly and independently associated with SRH even when controlling for physical illness and functional ability. Furthermore, anxiety/depression was more common among women than among men which may have contributed to women’s poorer SRH in the unadjusted model.

Previous studies have also shown that chronic pain in the musculoskeletal system belongs to the most common, costly, and disabling conditions in later life. Back-pain has been shown to be a common negative correlate of SRH [32] and musculoskeletal pain to be a major contributor to the burden of poor SRH in the population [7]. The effect of pain on SRH seems, however, to be more marked among younger age groups than older age groups [33]. In this study, almost eight out of ten persons reported to have at least some problems with pain. Pain was also one of the strongest factors for poorer than good SRH.

Vision impairment can lead to lower physical activity and reduced mobility [34]. In addition, communication problems, due to vision or hearing impairment, can lead to restricted social participation. For older people, hearing loss can lead to cognitive decline and impairment [34]. Both vision impairment and impaired hearing were associated with increased risk of poorer than good SRH in the bivariate models, but the associations were no longer statistically significant when other factors were taken into account. This disagrees with the findings of some other studies that have studied the association between vision and hearing impairment and SRH [19, 23].

About 10 % of the participants in our study had had an accident that led to seeking health care or dental care during the last 3 months. Accidents, especially falls, cause a substantial burden to patients and health care systems. Older adults who experience falls also report increased anxiety and depression and reduced quality of life [35]. No independent association was, however, found between accidents and SRH in our study. The interpretation of the term accident may vary between individuals which may have contributed to this lack of association.

Physical activity has been shown to be associated with SRH among elderly both in cross-sectional [36] and longitudinal studies [17, 18]. There is evidence that regular physical activity is safe for healthy and for frail older people and the risks of developing many health problems such as cardiovascular and metabolic diseases, obesity, falls, cognitive impairments, osteoporosis and muscular weakness are decreased by regular activities [37]. In our study, physical activity was associated with SRH in the bivariate analysis, and the association remained statistically significant - although weaker - after the adjustment for the other included determinants. This attenuation of the association may be due to that persons with impaired physical mobility or pain have more difficulties to be physically active than others. It should be noted that few respondents reported that they had moderate, regular exercise or regular exercise and training, so those physically active in this study were mostly those who responded that they spend more than 3 h a week in moderately strenuous activities (not shown).

We did not find any associations between other lifestyle factors than physical activity and SRH even though previous studies have found an association between e.g. smoking and SRH in the elderly [18, 19, 33]. The lack of association with smoking is possibly related to the small number of smokers (2%) in our study. We were not able to distinguish ex-smokers from non-smokers. Ex-smokers, especially those who have quitted smoking due to illness, have worse SRH than non-smokers [36]. An inverse association between alcohol use and SRH was found in the bivariate analysis, but this association was explained by other factors included in the adjusted model. Former drinking is associated with poor SRH [36] and older people with health problems are more prone to abstain from alcohol than those without [38], which may have contributed to this explanation. In line with some other studies an increased risk of poorer than good SRH was also observed for obesity [32, 33] but this association was no longer significant in the adjusted model.

No independent associations between SRH and living conditions such as educational level, country of birth, living alone, cash margin and social support were found, even though low educational level and lack of social support were associated with poorer than good SRH in the bivariate analyses. This contradicts the findings from some previous studies where for example education [20, 36], wealth and ethnicity [27] as well as social support [19] and loneliness [33] have been found to be associated with SRH in the elderly. However, some of these findings were obtained among younger elderly, whereas a smaller study on very old people, 80–89 years of age, in Sweden and Latvia did not find any association between SRH and educational level, income, marital status or social support [23] and one study in Spain found that the effect of social class among those aged 65 or older decreased with age [9].

### Strengths and limitations

The limitations of this study include the cross-sectional design, which precludes any interpretations about causality. In addition, the response rate was 47%. It is however common that the response rate is around 50% in population surveys [14], or even lower among very old people [23]. In Sweden, about 19% of people 80 years of age suffer from dementia and every third person 90 years of age or older [39]. Therefore, the survey did probably not reach or was not answered by the most ill or disabled persons and the prevalence of poor SRH may be underreported. On the other hand, it has been suggested that SRH is not associated with cognitive impairment among the oldest-old [15, 22]. It is therefore unlikely that the associations found in this study could be explained by non-response. Yet, the study is reflecting the national context in Sweden and includes only two counties. Another limitation regards the dichotomisation of the measures which might reduce the specificity of the data.

We did not collect data on the number of respondents who received help for filling in the questionnaire. In a later corresponding population survey the proportion of respondents who received help in this age group was considerable, 26% [40]. Receiving help for filling in the questionnaire may have generated some social desirability bias in our study. For example, estimates of positive health status and engaging in desirable behaviours have been found to be exaggerated when based on face-to-face or telephone interviews compared to self-administration methods [41].

One strength of the present study is that it is population-based and conducted among the elderly in their normal environment. The age-group studied, 85 years and older, is often excluded from population surveys or not studied separately from younger elderly. In addition, a broad set of factors describing living conditions and lifestyle factors as well as the most common health problems and functional ability in relation to SRH could be investigated. An additional strength is the use of SRH as health outcome, as it is a well-known and validated instrument [11, 42]. Pain and anxiety/depression were measured using EQ-5D, which is a standardized measure of health outcomes [26].

The population survey was conducted in enlarged samples in four counties in mid-Sweden in 2012 [43], but in only two counties it was extended to include the age group 85 years or older. This was done to explore whether the same survey questionnaire can be used in more advanced age groups than is the coverage in the national survey.

## Conclusions

Most of the participants in this study had at least fair self-rated health. The results are in line with the notion that, in the oldest-old, physical activity and such highly prevalent health problems as pain and depression as well as impaired physical mobility are important factors for self-rated health. This is useful information for policymakers and public health experts when considering how to improve and maintain health in the growing population of persons 85 years and older. Future studies investigating self-rated health and contributing factors among the oldest-old in the general population should examine different populations and, when possible, use longitudinal study design.

## Data Availability

The dataset analysed during the current study is not publicly available due to confidentiality and regulations under the Swedish law (the law of official statistics (2001:99 6§) and the law of secrecy (1980:100 9 chap. 4§)).
